# New Assay Systems to Characterize the Broad-Spectrum Antiherpesviral and Non-Herpesviral Activity of Cyclin-Dependent Kinase (CDK) 8 Inhibitors

**DOI:** 10.3390/ph18101560

**Published:** 2025-10-16

**Authors:** Debora Obergfäll, Friedrich Hahn, Jintawee Kicuntod, Christina Wangen, Melanie Kögler, Sabrina Wagner, Benedikt Kaufer, Manfred Marschall

**Affiliations:** 1 Harald zur Hausen Institute of Virology, Friedrich-Alexander-Universität Erlangen-Nürnberg (FAU), 91054 Erlangen, Germany; debora.obergfaell@fau.de (D.O.); friedrich.hahn@uni-ulm.de (F.H.); jintawee.kicuntod@uk-erlangen.de (J.K.); christina.wangen@uk-erlangen.de (C.W.); melanie.koegler@fau.de (M.K.); sabrina.wagner@uk-erlangen.de (S.W.); 2 Institute of Virology, Freie Universität Berlin, 14163 Berlin, Germany; benedikt.kaufer@fu-berlin.de

**Keywords:** human pathogenic viruses, α-, β-, γ-herpesviruses, antiviral drug discovery, novel host target proteins, cyclin-dependent kinase 8 (CDK8), pharmacological CDK8 inhibitors, pronounced antiviral activity, new antiviral assay systems, broad-spectrum antiviral drugs

## Abstract

**Background.** To date, a number of human pathogenic viruses are still unaddressed by the current repertoire of approved antiviral drugs. In order to widen this spectrum of preventive measures against virus infections, we have focused on additional host targets that exert interesting virus-supportive functions. Inhibitors of cyclin-dependent kinase 8 (CDK8) have been found to exhibit highly pronounced and relatively broad antiviral activity. **Objectives.** The current research question concerning the potential for broad-spectrum antiviral drug activity should be addressed in detail to understand the mechanistic basis of the antiviral target function of CDK8. **Materials and Methods.** We established and specifically customized six assay systems, three of these newly developed for the present study, to corroborate the range of CDK8 inhibitors’ antiviral activity against four α-, β-, and γ-herpesviruses as well as two non-herpesviruses. **Results.** Similar to our earlier analysis of CDK7 and CDK9 inhibitors, the clinically relevant CDK8 inhibitors currently in use demonstrated antiherpesviral activity in cell-culture-based infection models. Interestingly, the antiviral efficacy against various human and animal cytomegaloviruses was particularly strong at nanomolar concentrations, whereas other herpesviruses or non-herpesviruses showed an intermediate or low sensitivity to CDK8 inhibitors. Thus, this approach provided novel insights into the inhibitory potential of the CDK8 inhibitors, such as CCT-251921, MSC-2530818, and BI-1347, when analyzed against equine herpesvirus 1 (EHV-1, α-herpesvirus), human herpesvirus 6A (HHV-6A, β), Epstein–Barr virus (EBV, γ), murine herpesvirus 68 (MHV-68, γ), vaccinia virus (VV, non-herpes DNA virus), and severe acute respiratory syndrome coronavirus 2 (SARS-CoV-2, non-herpes RNA virus). **Conclusions.** Our results confirm that drug sensitivity to CDK8 inhibitors, on the one hand, is very strong for certain viruses and, on the other hand, varies widely within the spectrum of viruses and host cell types analyzed. This suggests that CDK8 may play several different roles in viral replication. The option of a refined CDK8-specific antiviral drug targeting is discussed.

## 1. Introduction

Antiviral drug research has become an actively processed field. Not only through the driving force of COVID-19 pandemic, but also through decades of intense viral and antiviral research, the repertoire of clinically available drugs and vaccines against human pathogenic viruses has markedly increased. Nevertheless, still only a small number of all relevant human viruses can be addressed by preventive and curative measures, so that additional novel approaches with so-far-unexploited mechanisms of inhibitors and innovative targeting strategies are urgently needed. This attempt may include both the conventional antiviral targeting with direct-acting antiviral drugs (DAAs) and the newly recognized chances with host-directed antiviral strategies (HDAs). In areas spanning the treatments of tumor, metabolic, infectious, and inflammatory diseases [1,2,3,4,5,6,7,8,9,10,11,12,13,14], HDAs already proved great success and strongly suggested their extended application into antiviral treatment. In this field of antiviral therapy, however, the topic misses in-detail experience, since only a low number of HDAs have been clinically approved and are in practical use (such as maraviroc, ribavirin, and bulevirtide), mostly against human immunodeficiency and hepatitis viruses, so far (references for HIV/maraviroc [15,16,17]; HCV/ribavirin [18,19,20,21,22,23]; HDV and HBV/bulevirtide [24,25,26,27,28,29]).

Our recent achievements in the field pointed to the very promising antiviral activity of protein kinase inhibitors. This new gain of knowledge did not only concern the special situation that herpesviruses encode their own protein kinases (HvPKs; [30]) and that the respective inhibitors comprise a pronounced antiviral potency, such as the clinically approved inhibitor of cytomegalovirus kinase pUL97, maribavir (MBV; [31,32,33]). In addition, this aspect also includes virus-supportive functions of cyclin-dependent kinases of the host (CDKs) and the previously reported potency of antiviral CDK inhibitors [34,35,36,37,38]. Moreover, based on the recognition that specific HvPKs can represent viral orthologs of host CDKs, i.e., vCDK/pUL97 [33,39,40], we fostered the understanding that inhibitors of host kinases, in ideal terms, CDKs and vCDKs, actually bear a huge potential of antiherpesviral cotreatment synergy [3,10,11]. Moreover, a specific point of relevance may be presented by the demonstration of a broad-spectrum antiviral activity of certain selective CDK inhibitors, such as those targeting CDKs 2, 7, 8, and 9 [35,41,42,43,44,45,46,47,48]. In particular, CDK8 is a complex regulator with manifold activities, as CDK8 recruits the core Mediator and super elongation complex (SEC) and is able to deactivate the CDK7–cyclin H–MAT1 transcription complex by phosphorylation [49]. Interestingly, these activities have recently been considered as rate-limiting steps of herpesviral replication efficiency, particularly of HCMV replication [38], but this CDK8 linkage also involves a number of other human and animal pathogenic viruses [35,46,50]. In the present study, our experimentation focused on the role of CDK8 as both a virus-supportive regulator and a putative novel antiviral drug target. To this end, we established new assay systems to assess the antiviral properties of clinically relevant CDK8 inhibitors.

As previously reported by other researchers, the three current inhibitors of interest exert a strong activity against human CDK8 [51,52,53]. They represent candidate drugs for ongoing clinical development. It should also be noted that secondary, lower-affinity targets of these CDK8 inhibitors have been identified, including CDK19 and potentially other CDKs [51,52,53]. As this is a general phenomenon of many CDK inhibitors that partly exert cross-talk with related kinases, the antiviral efficacy of these inhibitors may be based on a mode of action that goes beyond CDK8 mono-selectivity. Nevertheless, apart from this mechanistic aspect, the main target CDK8 has already been shown to be highly relevant for herpesvirus replication [46]. Thus, our current data contribute to specifying the broad-spectrum antiherpesviral and non-herpesviral activity of this type of host-directed antiviral.

## 2. Results and Discussion

### 2.1. Establishment of New Antiviral Assay Systems for a Selection of Human and Animal Pathogenic Viruses

In order to evaluate the postulated broadness on the antiviral efficacy of CDK8 inhibitors, new antiviral assay systems were established, including a selection of human and animal pathogenic viruses. Thus, α-, β-, and γ-herpesviruses as well as non-herpesvirus DNA and RNA viruses were utilized in parallel settings. These quantitative assay systems were either newly established or specifically optimized and provided a valuable platform for comparative antiviral drug analysis (Table 1). Notably, these assays, which were established and customized for EHV-1, HHV-6A, and VV, were applied for the first time in this study. The SARS-CoV-2 [54], MHV-68, and EBV assays were further developed in this study. The latter two, MHV-68 and EBV, have recently been used in brief form [35,48,55]. Of note is the fact that the various readout systems, which are mostly based on reporter expression, were applied using a number of different host cell types to assess the specific drug sensitivity of viral replication to CDK8 inhibitors and reference compounds. Such references, all possessing a previously characterized antiviral activity, were particularly useful as tools to optimize the respective virus replication models. The main focus was directed to the antiviral activity of three clinically relevant CDK8 inhibitors. Notably, the possibility that secondary targets recognized by these inhibitors (such as CDK19 [51,52,53]) play an accessory role cannot be fully ruled out. However, it is unknown whether the putative secondary targets of the present CDK8 inhibitors are relevant to herpesviral replication. Apart from this open question, the antiviral efficacy of these compounds, based on the main target CDK8, is undoubtedly strong. Thus, the sensitivity of the selected viruses to the inhibitors in these cell types could be categorized as strong, intermediate, low, or variable (Table 1).

### 2.2. Assessment of Antiviral Activity of Three Selected CDK8 Inhibitors Against α-, β-, and γ-Herpesviruses

**Figure 1:**
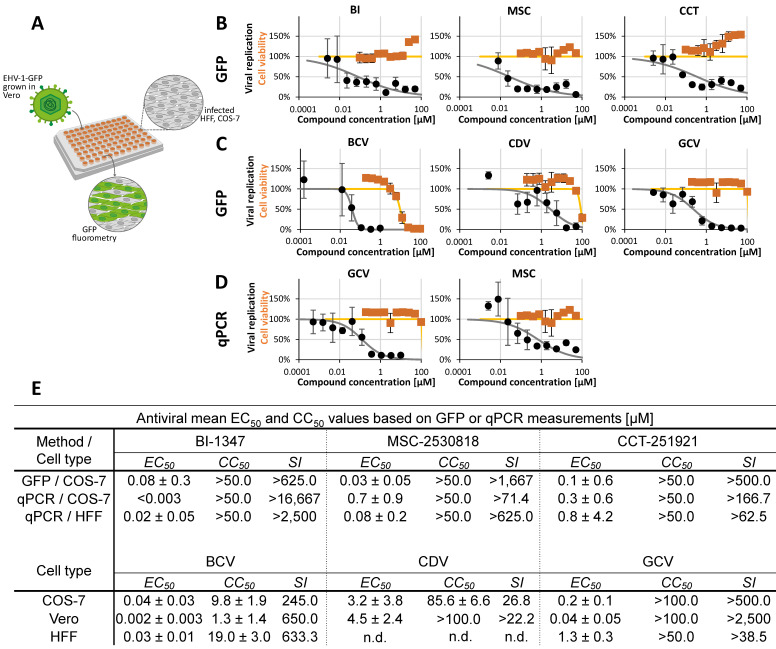
Determination of anti-EHV-1 activity and effects on cell viability, as exerted by CDK8 inhibitors and reference compounds. (**A**) Schematic illustration of the EHV-1-GFP antiviral assay. (**B**) CDK8 inhibitors (BI-1347, CCT-251921, MSC-2530818) were assessed for their level of cytotoxicity by a Neutral Red uptake assay (orange) and their antiviral activity (black) on COS-7 cells. Percentages of cell viability or viral replication were calculated and compared to DMSO treatment; mean values of triplicates ± SD are given. (**C**) Three reference compounds (GCV, CDV, BCV) were tested for their level of cytotoxicity (orange line, CC_50_) on COS-7 cells. The antiviral activity of reference compounds was measured by GFP approaches (black line, EC_50_). Percentages of cell viability or viral replication were calculated and compared to DMSO treatment; mean values of triplicates ± SD are given. (**D**) In addition, a qPCR approach was performed (GCV and MSC-2530818 shown on COS-7 cells). (**E**) Summarized results for different cell types (Vero, COS-7 and HFFs) or different method approaches (GFP or qPCR) for the CDK8 inhibitors (BI-1347, CCT-251921, MSC-2530818) or reference compounds (BCV, CDV, GCV) based on the EC_50_, CC_50_, and SI of at least three replications; n.d. not determined.

Concerning the α-herpesviruses, equine herpesvirus 1 (EHV-1) was chosen as a candidate for comparing inhibitory drug profiles between infected-cell cultures because it can infect a number of different animal hosts, including horses, cattle, and rodents. It may also be useful as an in vivo mouse model in the future [56,57]. For our analyses, we established an EHV-1 GFP-based assay. To this end, we infected 96-well plate cultures of Vero, COS-7, MDCKII, HFFs, or MEFs with a titer-determined stock of EHV-1-GFP. The first readout was GFP-specific automated fluorometry (VictorX4; Table 1). Reliable levels of GFP signals were obtained for EHV-1-GFP infection of Vero, COS-7 cells, or HFFs, whereas only very low signal levels were obtained for MEFs or MDCKII cells. To confirm sufficient EHV-1 permissiveness, a virus-specific qPCR was performed on samples of infected cell culture media. Based on the intensity of these signals, the permissiveness of the cells to EHV-1 could be ranked as follows: COS-7 > HFF > Vero > MDCKII > MEF (MDCKII and MEF were basically not susceptible to EHV-1 infection). Therefore, COS-7 cells were primarily selected for detailed analysis (Figure 1). The antiviral system was adjusted using reference drugs, i.e., GCV, CDV, and BCV (Figure 1C), and readouts were performed using reporter GFP measurements with infected cell layers and EHV-1-specific qPCR based on virus release into the culture medium (Figure 1D). Concentration-specific curves of antiviral activity were obtained in all cases, with half-maximal effective mean concentrations (EC_50_ values) ranging from 0.002 ± 0.003 µM to 4.50 ± 2.40 µM. The anti-EHV-1 efficacies of CDK8 inhibitors were pronounced, with submicromolar concentrations (Figure 1B). The three compounds BI (BI-1347), CCT (CCT-251921), and MSC (MSC-2530818) were compared based on GFP and qPCR measurements (Figure 1E). The strongest inhibitory efficacy was found for BI in EHV-1-infected COS-7 cells, with EC_50_ values down to the nanomolar range. Interestingly, the EHV-1-directed efficacy of the three CDK8 inhibitors showed some quantitative variation in HFFs (GFP measurement; Figure 1E), with EC_50_ values still in a submicromolar range. These differences in the efficacy of identical compounds against one specific virus in different host cell types (see further examples below) may reflect the host-directed antiviral mode of these compounds. This may point to a cell-type-specific inhibitory mode, such as virus activation, maturation, release, etc. Specifically, the virus-supportive CDK8 target may exhibit differential expression levels in various cell types, which may be functionally compensated, at least in part, by cell-type-specific expression of related CDKs.

**Figure 2:**
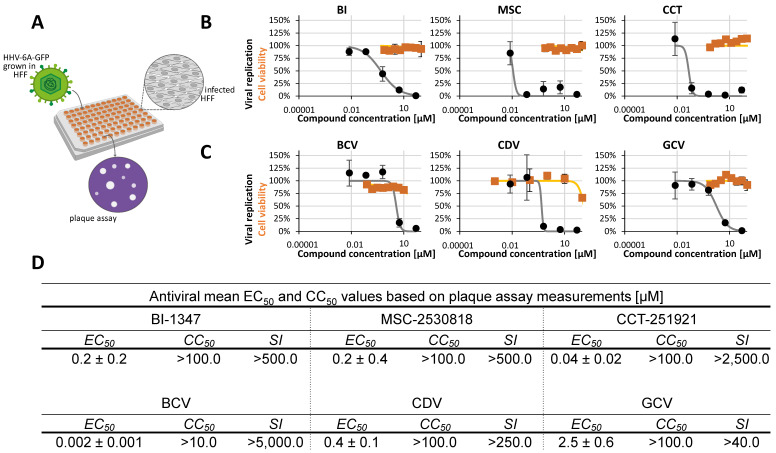
Determination of anti-HHV-6 activity under newly established conditions of plaque reduction assay using primary human fibroblasts. (**A**) Schematic representation of the antiviral assay used to evaluate compound activity against HHV-6A. (**B**) CDK8 inhibitors (BI-1347, CCT-251921, MSC-2530818) were analyzed for cytotoxic effects using a Neutral Red uptake assay (orange lines) and for antiviral activity using a plaque assay (black lines). Cell viability and viral replication are expressed as percentages relative to DMSO-treated controls. (**C**) Reference compounds (GCV, CDV, BCV) were evaluated for cytotoxicity (orange lines) and for antiviral efficacy (black lines). Results are presented as percentages relative to DMSO treatment and shown as mean ± SD of triplicates (NRA) and duplicates (plaque assay). Each plaque assay plate was counted twice. (**D**) Summary of EC_50_, CC_50_, and selectivity index (SI) values for CDK8 inhibitors and reference compounds based on at least three independent experiments.

Concerning the β-herpesviruses, human herpesvirus 6A (HHV-6A) was used because it is a close relative of cytomegaloviruses (CMVs) that is moderately pathogenic. It should be compared to CMVs in terms of sensitivity to antiviral CDK inhibitors, which had been analyzed against various CMV strains and reporter recombinants in detail before [11,35,43]. To this end, HHV-6A-GFP was used to infect the primary fibroblast model of HCMV infection (HFFs), and an unachieved readout was established in these cells using a plaque reduction assay (PRA; Table 1). This newly established PRA-based measurement of anti-HHV-6A drug activity in HFFs is specifically improved and useful because it allows for fresh infection with an HHV-6A virus stock instead of using HHV-6A-positive J-Jhan carrier cells, which carry HHV-6A genomes in an integrated form and continuously produce progeny virus. Based on our experience with this method of drug assessment in HFFs, we observed that HHV-6A is more sensitive with this fresh infection and PRA readout (Figure 2). We adjusted the assay conditions using antiherpesviral reference drugs, i.e., BCV, CDV, and GCV (Figure 2C). Regarding the CDK8 inhibitors, we reconsidered our previous statement that HHV-6A has very limited sensitivity to these types of HDAs [35]. Instead, we recognized the clear anti-HHV-6A efficacy of all three analyzed compounds under these assay conditions (Figure 2B). The mean EC_50_ values of BI, CCT, and MSC in the HFF infection system were 0.2 ± 0.2, 0.2 ± 0.4, and 0.04 ± 0.2 µM, respectively.

**Figure 3:**
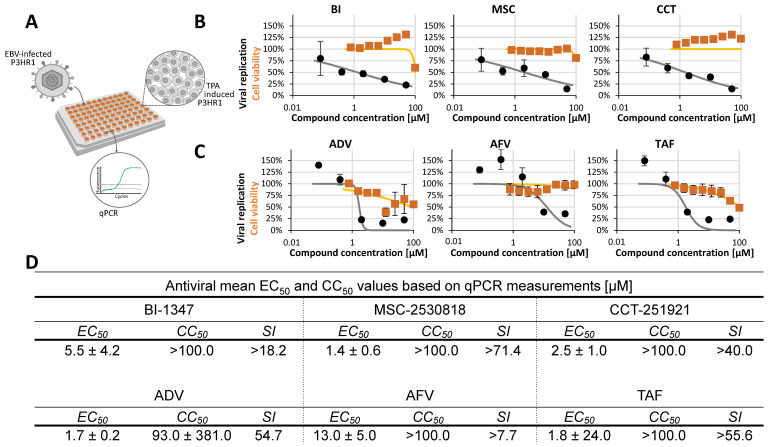
Determination of anti-EBV activity in lytic assay system with TPA-induced P3HR-1 cells. (**A**) Schematic overview of the antiviral assay designed to assess compound activity against EBV. (**B**) CDK8 inhibitors (BI-1347, CCT-251921, MSC-2530818) were tested for cytotoxicity using a Neutral Red uptake assay (orange line) and for antiviral efficacy by qPCR (black line). Cell viability and viral replication are expressed as percentages relative to DMSO-treated controls. (**C**) The reference compounds, adefovir dipivoxil (ADV), adefovir (AFV), and tenofovir alafenamide (TAF), were evaluated for their cytotoxic effects (orange lines) and for antiviral activity (black lines). Data represent mean ± SD of three replicates normalized to DMSO controls. (**D**) Summary of EC_50_, CC_50_, and selectivity index (SI) values for CDK8 inhibitors and reference compounds based on a minimum of two independent experiments.

Concerning the γ-herpesviruses, Epstein–Barr virus (EBV) was chosen, as it represents a major human oncogenic viral pathogen with worldwide clinical importance. EBV was specifically included in the comparative CDK inhibitor analysis because well-established quantitative anti-EBV drug research systems are rare. Therefore, we further developed the lytic EBV system with TPA-induced P3HR-1 cells. The productive viral replication and release could be quantified by qPCR detection of EBV genomic loads in culture media samples of a 96-well plate format (Table 1). The antiviral data indicate an intermediate sensitivity of EBV replication to CDK8 inhibitors. As based on the P3HR-1 model, EC_50_ values were obtained in the range of 5.5 ± 4.2, 1.4 ± 0.6, and 2.5 ± 1.0 µM for the three compounds (Figure 3).

**Figure 4:**
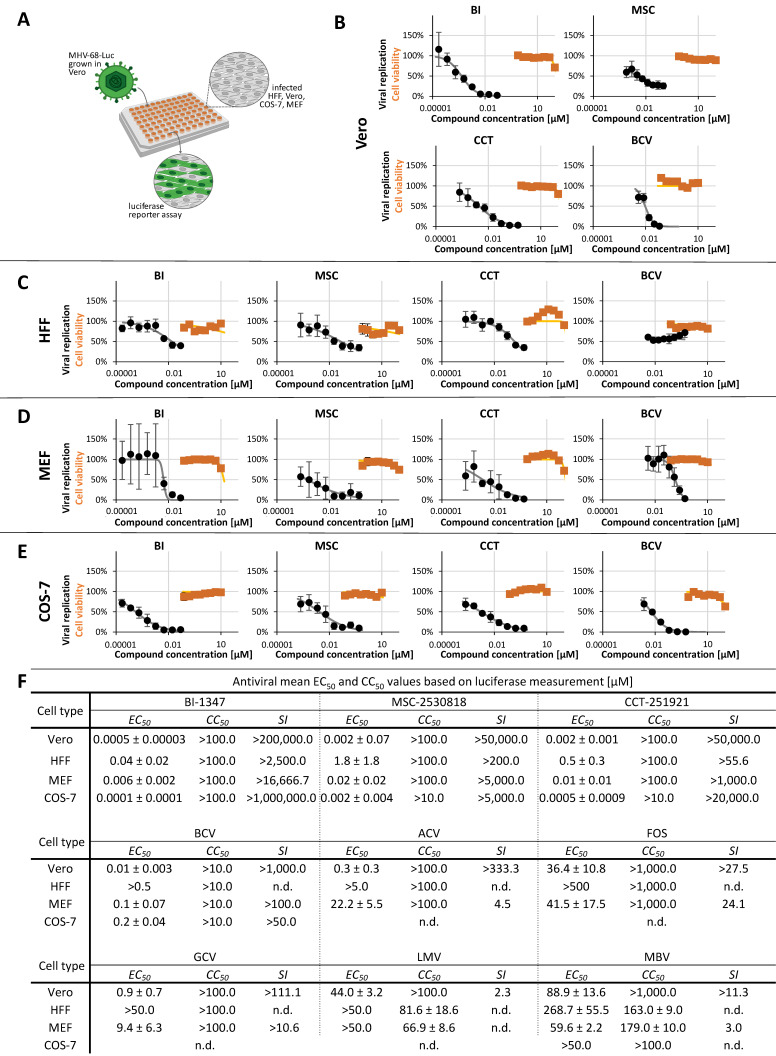
Determination of anti-MHV-68-Luc activity by the use of a luciferase-based assay, established in four different animal and human cell types. (**A**) Schematic illustration of the MHV-68-Luc antiviral assay. CDK8 inhibitors (BI-1347, CCT-251921, MSC-2530818) and BCV were tested for cytotoxicity using a Neutral Red uptake assay (orange line) and for antiviral efficacy by a luciferase assay (black line) in different cell types: (**B**) infected Vero cells, (**C**) HFFs, (**D**) MEFs, and (**E**) COS-7 cells. Cell viability and viral replication are expressed as percentages relative to DMSO-treated controls for three (NRA) and four (luciferase assay) replicates. (**F**) Summary of EC_50_, CC_50_, and selectivity index (SI) values for CDK8 inhibitors and reference compounds (ACV, BCV, FOS, GCV, LMV, and MBV) for different cell types (Vero, COS-7, HFF, and MEF) based on a minimum of three independent experiments; n.d. not determined.

In addition to EBV, MHV-68-Luc, a murine model virus, was applied. The Luc reporter signal, provided by MHV-68-Luc infection, proved to be a reliable quantitative marker of virus replication and release (Figure S1). During the refinement of this virus system, MHV-68-Luc demonstrated an enhanced ability to undergo productive replication in various host cell types. Consequently, the analysis of host-directed antiviral CDK8 inhibitors yielded valuable insights. Here, we compared anti-MHV-68 drug efficacy in four different cell types, i.e., Vero, MEF, HFF, and COS-7 cells, to verify and specify our previous finding of a particularly strong anti-MHV-68 activity of BI, CCT, and MSC (Figure 4) [35]. Our current findings confirm the nanomolar-range activity of CDK8 inhibitors against MHV-68-Luc and provide additional data for MHV-68-Luc in independent cell systems. Finally, our findings specify the antiviral potency as directly dependent on the infected host cell type. In particular, regarding the latter aspect, we substantiated our statement about the importance of cell type for the antiviral CDK8 targeting. Here, three factors may be relevant for MHV-68 in cell-type comparison, namely, the species varieties of CDK8, the differential CDK8 expression levels in cell types, and the functional complementation between related CDKs.

### 2.3. Antiviral Activity of CDK8 Inhibitors Across Herpesviral Subfamilies

**Figure 5:**
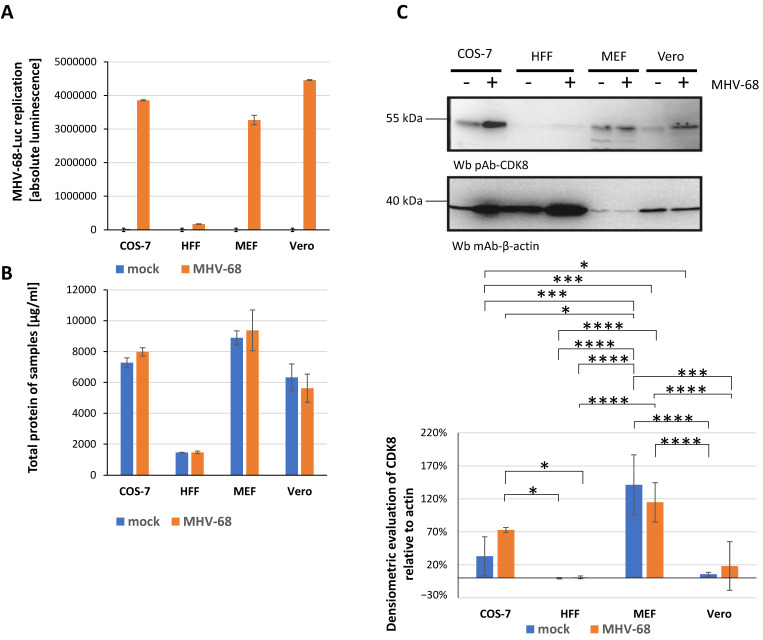
Comparison of expression levels of CDK8 in different MHV-68-permissive host cell types. MHV-68-Luc-infected cells were harvested 4 d p.i. and lysed for further analysis. (**A**) Luciferase assay performed as MHV-68-Luc infection control for COS-7, HFF, MEF and Vero cells. The columns of uninfected (mock) samples are displayed at the left (blue) and the MHV-68-Luc-infected samples at the right (orange). (**B**) BCA assay values are displayed as measures of total cellular protein content. (**C**) Total protein lysates (30 µg/mL) were prepared from uninfected (mock/−) or MHV-68-infected (MHV-68-Luc/+) cells and subjected to standard SDS-PAGE/WB procedures. CDK8 levels were detected by Wb staining using mAb-CDK8; β-actin was stained as a loading control. Based on the WB analysis, a densiometric determination, using AIDA, was performed for CDK8 levels relative to actin. Analysis was performed in quadruplicate measurements (SDS-PAGE/Wbs in duplicate, densitometry in duplicate), and statistical evaluation was carried out using ANOVA followed by Bonferoni’s correction for multiple comparisons. *, *p* ≤ 0.05; ***, *p* ≤ 0.001; ****, *p* ≤ 0.0001.

In a previous report, we could already identify a marked antiviral efficacy of CDK8 inhibitors against several, but not all, of the investigated herpesviruses [35]. In the present study, we used additional α-, β-, and γ-herpesviruses to address their sensitivity towards the three CDK8 inhibitors of current interest. Findings from the newly adopted assay systems for EHV-1, HHV-6A, EBV, and MHV-68 (see Table 1 and Table S1) revealed that the drugs BI, CCT, and MSC have broad antiherpesviral activity. In general, CDK8 appears to be an important virus-supportive host factor; however, the magnitude of CDK8 inhibitor sensitivity varied significantly among the herpesvirus species. A novel finding of this study is that the impact of host cell types is at least as important as the analyzed virus species. A novel finding of this study is that the impact of host cell types is at least as important as the analyzed virus species. This was evident when three or four different host cells were used for EHV-1 and MHV-68, respectively. For both viruses, the EC_50_ values of the three drugs varied depending on the infected cell type. This may be due to several reasons, as discussed briefly in the previous section. The main reason may be related to cell-type-specific expression characteristics of CDK8 and other CDKs that can provide cross-complementing virus-supportive functionality. A very marked example was given by MHV-68 because, here, the difference in sensitivity to compound BI (CCT/MSC) was specifically high when comparing the infection of Vero, COS-7, HFF, or MEF, i.e., primate, human, and murine cell types (Figure 4F). Such a difference in cell-type-specific CDK8 expression levels could be illustrated by semi-quantitative Wb analysis (Figure 5). These results indicated that the expression levels of CDK8 varied substantially in the MHV-68-permissive host cell types (as based on the cross-species reactivity of the CDK8-specific antibody used for all Wb stainings). Thus, variability in anti-viral CDK8 inhibitor sensitivity may reflect differences in virus–host interactions. Among these differences, quantitative and qualitative differential levels of host CDKs appear relevant. Apart from individual variations, we confirmed the broad-spectrum activity tendency of CDK8 inhibitors across representatives of α-, β-, and γ-herpesviruses (see summarizing Table S1, comparing data of the present study with those previously reported). Therefore, CDK8 could be considered a target for the next generation of host-directed antiviral drug development.

### 2.4. Addressing the Characteristics of Antiviral MoA Displayed by CDK8 Inhibitors Against Three Strains of HCMV: Time-of-Addition Experimentation

**Figure 6:**
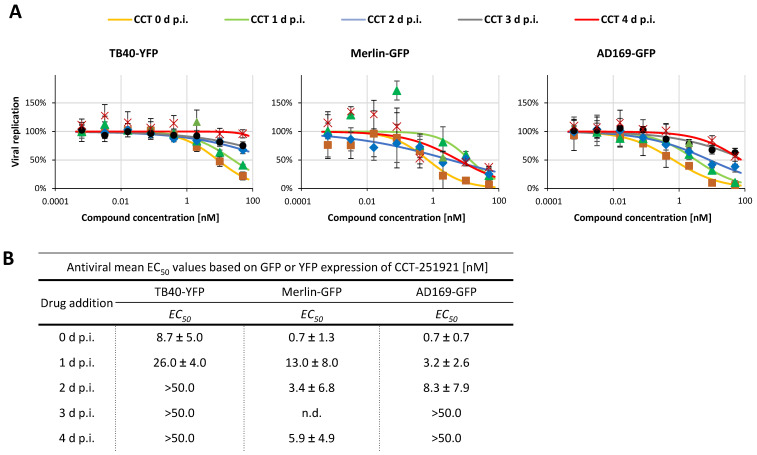
Determination of anti-HCMV activity at different time points of treatment onset. (**A**) Evaluation of antiviral treatment with CCT-251921, determined for HFFs infected with HCMV TB40-YFP, Merlin-GFP, or AD169-GFP. The compound was added at the indicated time point of infection (0 d p.i., orange curves), 1 d p.i. (green), 2 d p.i. (blue), 3 d p.i. (grey), and 4 d p.i. (red); reporter measurement and the determination of EC_50_ values was performed at 7 d p.i. in all cases. Viral replication is shown as percentages relative to DMSO-treated controls of four replicates. (**B**) Summary of EC_50_ values of CCT-251921 after onset of treatment at different time points (0 d p.i. to 4 d p.i.), determined for three different HCMV strains; n.d., not determined.

The first characteristics of the anti-HCMV-directed mode-of-action (MoA) of CDK8 inhibitors have been addressed by previous investigations by our group [35,38,58]. Thereby, a marked late-phase inhibitory activity of pharmacological CDK8 inhibition by CCT as well as siRNA-mediated CDK8 knock-down could be identified [35]. In the current context of analyses, we addressed the question of antiviral efficacy of CCT upon delayed drug addition to a multi-round infection setting. To his end, HFFs were infected with strains of HCMV before the CDK8 inhibitor CCT was applied for antiviral treatment (serial 5-fold dilutions at concentrations of 50 nM to 0.00064 nM) at the time points indicated (0 d p.i. to 4 d p.i.). Three different viral strains (expressing YFP or GFP reporter as indicated) were used for infection, i.e., the tropism-adaptable HCMV TB40, the genetically intact clinical isolate HCMV Merlin, and the laboratory HCMV strain AD169 (Figure 6). The strains Merlin and AD169 showed a particularly strong sensitivity to CCT inhibition (at EC_50_ concentrations compatible with earlier investigations; [35]), and strain TB40 showed a lower level of drug sensitivity (Figure 6A), which may be explained by the generally prolonged replication behavior of TB40 in this antiviral system [58]. The time-of-addition experiment in this multi-round replication assay indicated a requirement of early onset of drug treatment (starting at 0 d, 1 d, or 2 d p.i.), as clearly seen for the strains TB40 and AD169, respectively (Figure 6A,B, left and right). An addition of the drug at days 3 or 4 p.i. did no longer exert measurable antiviral activity for these two strains. The result suggests that CCT starts its host-directed antiviral effect at early time points of viral replication. The strain Merlin showed a slightly different temporal course of drug sensitivity, as here, the lowest EC_50_ values were also obtained under condition 0 d p.i., but even later time points of drug addition (up to 4 d p.i.) produced measurable antiviral efficacy (Figure 6A,B, central; quantitative variations in EC_50_ values might refer to an onset of viral cytopathic effects ≥ 2 d p.i.). This may indicate a specifically strong CDK8 dependency of the clinically relevant, genetically intact strain Merlin. Combined, the time-of-addition experiment provided further information on the antiviral MoA of the CDK8 inhibitor, in that, on the one hand, inhibitory activity is provided for various HCMV strains. On the other hand, drug addition must be ensured during the early period (days 0–2 p.i.) of the first HCMV replication cycle, which lasts 3–4 days within a multi-round replication assay (terminated at day 7 p.i.).

### 2.5. Non-Herpesviral Activities of CDK8 Inhibitors

**Figure 7:**
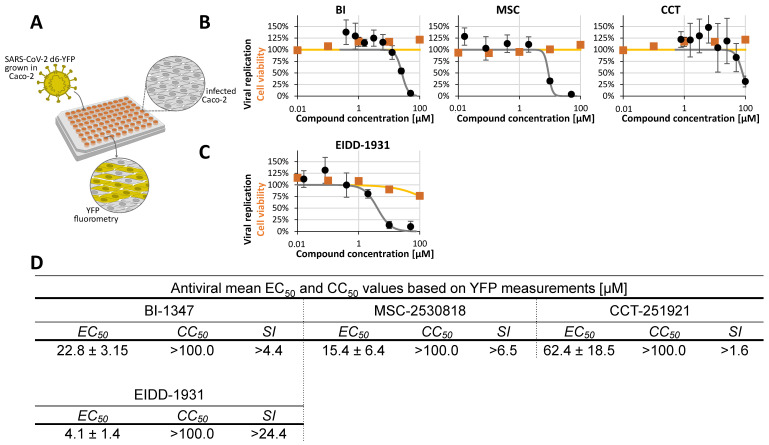
Determination of anti-SARS-CoV-2 activity using the reporter virus d6-YFP in Caco-2 cell infection. (**A**) Schematic illustration of the SARS-CoV-2 d6-YFP antiviral assay setup. (**B**) CDK8 inhibitors (BI-1347, CCT-251921, MSC-2530818) were evaluated for cytotoxicity using a Neutral Red uptake assay (orange line) and for antiviral activity based on YFP expression (black line). Cell viability and viral replication are shown as percentages relative to DMSO-treated controls. (**C**) The reference compound EIDD-1931 (the active form of molnupiravir/MPV) was assessed for cytotoxicity as a comparative control drug. Data represent mean ± SD from four replicates normalized to DMSO controls. (**D**) Summary of EC_50_, CC_50_, and selectivity index (SI) values for the CDK8 inhibitors and the reference compound based on at least two independent experiments.

In order to further address the question of a broader antiviral potency of CDK8 inhibitors, we also analyzed two non-herpesviruses, i.e., VV (DNA virus) and SARS-CoV-2 (RNA virus), respectively. Using the assay systems developed and refined in this study, additional data could be collected for antiviral reference compounds in general and for CDK8 in particular. For SARS-CoV-2, the recently established reporter virus d6-YFP [54] was used for infection of Caco-2 cells in a 96-well plate (Figure 7A). Antiviral-drug-specific inhibition of lytic virus replication was quantified at 30 h p.i. by automated YFP-based fluorometry. Data indicate a very limited efficacy of CDK8 inhibitors against SARS-CoV-2 in this infection model (comprising low drug sensitivity expressed by the high micromolar EC_50_ values in a range between 15.4 and 62.4 µM, Figure 7D). Thus, our data indicated a lack of CDK8 inhibitor sensitivity (CCT) or a low level of sensitivity (BI, MSC) of SARS-CoV-2 in Caco-2 cells. None of the three compounds, i.e., BI, MSC, or CCT, produced an EC_50_ value lower than 15.4 µM (Figure 7B; compared to the EC_50_ value of 4.1 ± 1.4 µM for the control drug EIDD-1931/MPV, Figure 7C,D). However, it should be emphasized that the anti-SARS-CoV-2 activity of host-directed compounds may be virus-strain- or cell-type-specific [59]. This aspect may be relevant to our experimental system since the virus strain used to introduce the YFP reporter module was originally isolated from a patient in 2021 [54,60] and is likely not circulating naturally at present.

**Figure 8:**
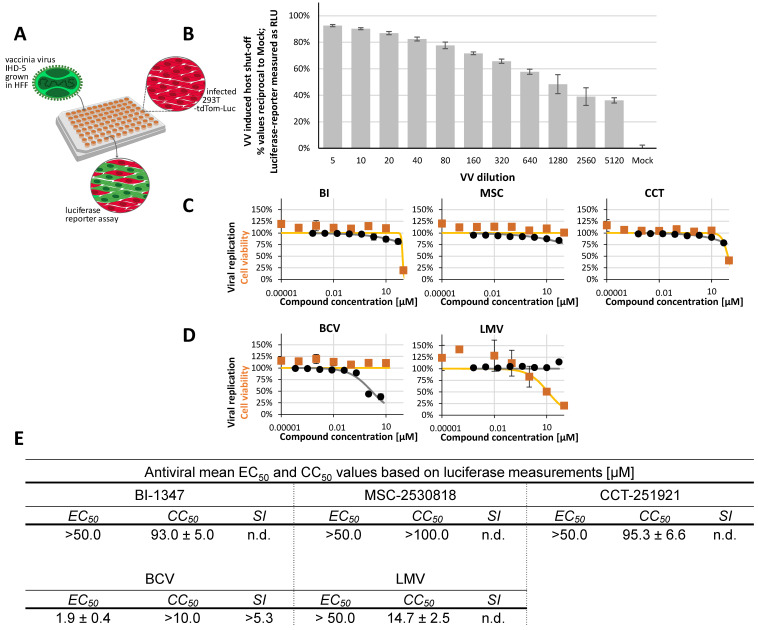
Determination of anti-VV activity using a luciferase-/infection-based reporter model. (**A**) Schematic illustration of the VV antiviral assay setup. (**B**) Titration of VV on 293T-tdTom-Luc to analyze dose-dependent virus-induced host shut-off based on luciferase expression. (**C**) CDK8 inhibitors (BI-1347, CCT-251921, MSC-2530818) were evaluated for cytotoxicity using a Neutral Red uptake assay (orange line) and for antiviral activity based on the VV-induced shut-off of the reporter cell line (293T-tdTom-Luc) (black line). Cell viability and viral replication are shown as percentages relative to DMSO-treated controls. (**D**) The reference compounds, BCV and LMV, were assessed for cytotoxicity (orange line) and antiviral efficacy (black line). Data represent mean ± SD from four replicates normalized to DMSO controls. (**E**) Summary of EC_50_, CC_50_, and selectivity index (SI) values for the CDK8 inhibitors and the reference compounds based on at least three independent experiments.

A highly sensitive reporter assay was developed for VV analysis, which detects VV-mediated shutdown of reporter protein expression. A 293T cell line stably expressing tdTom-Luc [61] served as a reporter model in a 96-well format to utilize VV-induced shutdown for monitoring antiviral drug efficacy VV infection correlated with complete luciferase activity in uninfected cells (Table 1). EC50 values reflected the antiviral rescue of VV-induced shutdown. The inhibitory effect of the reference compound BCV demonstrated the antiviral efficacy of VV replication at an EC50 of 1.9 ± 0.4 µM (Figure 8). Similar EC_50_ values for BCV in a low micromolar range were previously reported [62]. However, none of the three analyzed CDK8 inhibitors produced a measurable anti-VV activity in this system. This point adds to our earlier understanding that the antiviral efficacy of CDK8 inhibitors can be very high depending on the virus species and cellular environment. Thus, the findings of Figure 7 and Figure 8 support the earlier statement that the CDK8-controlled Mediator transcription complex can act as a virus-supportive factor for several human viruses [46], which is particularly relevant for human and animal herpesviruses but to a lesser extent for unrelated viruses.

## 3. Materials and Methods

### 3.1. Cells and Viruses

293T, Vero, COS-7, MEF, and MDCKII cells, all derived from ATCC (Manassas, VA, USA), were cultivated in Dulbecco’s modified Eagle’s medium (DMEM) (Thermo Fisher Scientific, Waltham, MA, USA) supplemented with 10% fetal bovine serum (FBS; Capricorn, Ebsdorfergrund, Germany), 1× GlutaMAX™ (Thermo Fisher Scientific, Waltham, MA, USA), and 10 μg/mL gentamicin (Thermo Fisher Scientific, Waltham, MA, USA). For Caco-2 cells (ATCC; Manassas, VA, USA), 1× non-essential amino acids (Thermo Fisher Scientific, Waltham, MA, USA) were added to the DMEM medium described above. Primary human foreskin fibroblasts (HFFs, clinical samples from the Children’s Hospital Erlangen, Germany) and murine embryonic fibroblasts (MEFs; ATCC, Manassas, VA, USA) were cultured in Eagle’s Minimal Essential Medium (MEM), and J-Jhan (kindly provided by Benedikt Kaufer, FU Berlin, Germany) and P3HR-1 B cells containing a lytic mutant of the EBV genome (kindly provided by Dr. Susanne Delecluse, DKFZ, Heidelberg, Germany, and Hans Helmut Niller, Virology, Univ. Regensburg, Germany) were cultured in Roswell Park Memorial Institute (RPMI) 1640 medium (Thermo Fisher Scientific, Waltham, MA, USA). All media were supplemented with 10% FBS, 1× GlutaMAX™, and 10 μg/mL gentamicin. Cells were maintained at 37 °C, 5% CO_2_, and 80% humidity and were regularly monitored for the absence of mycoplasma contamination using a Lonza™ Mycoalert™ kit (Thermo Fisher Scientific, Waltham, MA, USA). Viruses used in this study were equine herpesvirus 1 [63], human herpesvirus 6A (HHV-6A; infected J-Jhan/HHV-6A producer culture or BACmid-derived reconstituted virus stock; [64]), Epstein–Barr virus (EBV, strain P3HR-1, in immortalized P3HR-1 B cell producer culture), vaccinia virus (VV, strain IHD-5; American Type Culture Collection; [43]), and severe acute respiratory syndrome coronavirus 2 [54].

### 3.2. Antiviral Compounds

BI-1347, CCT-251921, MSC-2530818 targeting CDK8, adefovir (AFV), brincidofovir (BCV), cidofovir (CDV), EIDD-1931, ganciclovir (GCV), tenofovir alafenamide (TFA), and tenofovir disoproxil (TDP) as reference compounds were obtained from MedChemExpress, Monmouth Junction, NJ, USA. Stock solutions were prepared in aliquots using sterile DMSO (Sigma Aldrich, St. Louis, MO, USA) and stored at −20 °C.

### 3.3. Virus Infection of Cultured Cells and Quantitative Readouts of Viral Replication

Equine herpesvirus 1 (EHV-1). Various cell types were used for infection with EHV-1-GFP and analyzed for their virus-productive permissiveness. After 90 min of viral adsorption, the antiviral compounds were added to the infected cells without removing the viral inoculum. Cells were cultivated at 37 °C for 3 to 5 d depending on their permissiveness. Thereafter, cells were fixed with 10% formalin at room temperature for 10 min. The EHV-1-GFP signals were measured utilizing a Victor X4 microplate reader (VictorX4; PerkinElmer, Waltham, MA, USA).

Human herpesvirus 6A (HHV-6A). The HHV-6A-GFP genomic BACmid was purified with a NucleoBond Xtra BAC Kit (Macherey-Nagel, Düren, Germany) and used for virus reconstitution from transfected HFFs (Lipofectamine 3000; Thermo Fisher Scientific, Waltham, MA, USA) to obtain a cell-free HHV-6A stock. Based on this virus stock used as an inoculum, a classical plaque reduction assay was newly established for lytic HHV-6 infection of HFFs. For this, 1.5 × 10^4^ HFFs were seeded per well (24-well plates) 1 d prior to infection (MOI of 0.0005). After 90 min of virus adsorption, medium supernatants were removed, and the cells were treated with antiviral compounds or solvent control using 2× MEM (Thermo Fisher Scientific, Waltham, MA, USA). These samples were added to a heat-dissolved agarose 0.6% solution in 1:1 mixtures of medium–agarose (final 0.3%, partly cooled when adding) for further cultivation of cells. At 10 to 11 d p.i., the agarose overlay was removed before cells were stained with 1% crystal violet in 20% EtOH to count virus-induced plaque formation under a microscope.

Human cytomegalovirus (HCMV). Antiviral replication assays for HCMV reporter viruses were performed as described previously [35,65]. Briefly, HFFs were seeded in the 96-well format and infected with either AD169-GFP, TB40-YFP, or Merlin-GFP. To address the question of a time-dependent effect of CDK8 inhibitors, CCT-251921 was added at different time points post-infection (at 0 d, 1 d, 2 d, 3 d, or 4 d p.i.). Cells were harvested 7 d p.i. (or 12 d p.i. for Merlin-GFP) before GFP or YFP expression was measured using the Victor X4.

Epstein–Barr virus (EBV). The determination of anti-EBV drug activities was briefly described by our group previously in principle terms [43,55,66]. Here, we optimized the setup by using the EBV-positive immortalized B cell line P3HR-1, which is sensitive to lytic-cycle EBV production, as chemically induced with 40 ng/mL of 12-O-tetradecanoylphorbol-13-acetate (TPA) [55]. P3HR-1 cells were seeded in 96-well plates and were simultaneously induced with TPA and treated with antiviral compounds for 10 d before cells were subjected to quantitative assessment of EBV lytic productivity in the culture medium samples. Thus, at 10 d post-treatment, samples were exposed to proteinase K digestion, followed by the EBV/BGLF5-specific quantitative polymerase chain-reaction (qPCR) analysis (primers 5′-TGA CCT CTT GCA TGG CCT CT-3′ and 5′-CCT CTT TTC CAA GTC AGA ATT TGA C-3′; FAM probe 5′-CCA TCT ACC CAT CCT ACA CTG CGC TTT ACA-3′).

Murine gammaherpesvirus 68 (MHV-68). The reporter virus MHV-68-Luc was multiplied in Vero cells, and stock virus was used for antiviral drug assessment in a 96-well plate setting, as described before [35]. In brief, MHV-68-Luc-infected cells were incubated for the cell-type-specific durations indicated, lysed using 100 µL of lysis buffer per well, and applied to reporter luminescence analysis with sample volumes of 50 µL per measurement (0.1 M KH_2_PO_4_, 15 mM MgSO_4_, 5 mM ATP, 1 mM D-luciferin), as measured in the Perkin Elmer Victor X4 reader.

Vaccinia virus (VV). The VV-mediated host shut-off was determined using a specific reporter plasmid (kindly provided by Kazuhiro Oka [61]). For this, 293T cells stably expressing Luc2-P2A-tdTom were prepared by using a lentiviral gene transfer approach utilizing the reporter plasmid pCDH-EF1-Luc2-P2A-tdTom [67]. Positive transfected cells were selected with FACS-based cell sorting according to the tdTom expression. For the VV host shut-off assay, 2 × 10^4^ 293T-tdTom-Luc cells were seeded per well (96-well plate), before virus-medium inocula as well as antiviral compounds were gently added on the next day (to minimize 293T cell detachment). At 2 d p.i., cells were lysed in 100 µL of standard assay buffer (100 mM phosphate buffer (a combination of 1 M KH_2_PO_4_ and 1 M K_2_HPO_4_ to reach pH 7.8) and 15 mM MgSO_4_ supplemented with 1% Triton X-100) per well [58]. To quantify the luciferase (Luc) signal, 10 mM rATP (Abcam, Cambridge, UK) and 1 mM luciferin (PJK GmbH, Kleinbittersdorf, Germany) were added to the standard assay buffer. A volume of 50 μL of freshly prepared buffer was added to measure Luc signals using the Victor X4 microplate reader (VictorX4). Wells infected with VV and solvent-treated DMSO were used as a background (indicating a maximal VV-induced Luc signal shut-off), where mock-infected 293T-tdTom-Luc cells defined the 100% Luc level.

Severe acute respiratory syndrome coronavirus 2 (SARS-CoV-2). A SARS-CoV-2 replication assay was performed as previously described [54]. In brief, SARS-CoV-2 infection of Caco-2 cells was performed with the d6-YFP reporter virus in a 96-well plate format. Cells were harvested at 30 h p.i., and the quantities of viral replication were assessed by YFP quantitation in the Victor X4 microplate reader. Antiviral efficacy (mean of biological quadruplicates) was expressed as the percentage of the mock-treated control.

Concerning the fluorescence-based readouts, for EHV-1-GFP, HCMV AD169-GFP, and SARS-CoV-2 d6-YFP [35,48,54,58,68], the automated fluorometry measurements using the Victor X4 microplate reader (VictorX4) were compared with automated microscopy measurements using a Pico ImageXpress^®^ Device (PicoMD; Molecular Devices LLC, San Jose, CA, USA). Parallel measurements in the experiments provided confirmation of the data; however, in the context of this study, VictorX4 runs proved to be more facile and reliable, so these data are presented exclusively.

### 3.4. Neutral Red (NR) Uptake and Alamar Blue (AB) Cell Viability Assays

Uninfected cells (mock) were seeded at the same density as used in antiviral replication assays and treated with either test compounds or a solvent control. Durations of compound treatment were likewise adjusted to the periods in the antiviral assays in the following manner: HFFs, 7–14 d; Caco-2 cells, 3 d; 293T, 2 d; Vero, 7 d; COS-7, 4 d; MEF, 7 d; MDCKII, 7 d; J-Jhan, 14 d; P3HR-1, 10 d. At the end of the treatment period, Neutral Red (NR) was added to a final concentration of 40 μg/mL and incubated for 2–4 h at 37 °C. The NR-containing supernatant was finally removed, and cells were destained using a solution of 50% ethanol, 1% acetic acid, and 49% distilled water. For 293T cells, an Alamar Blue (AB) assay was alternatively used to assess cell viability at 2 d. Here, cells were seeded similarly and incubated with 25 ng/mL resazurin (AB; Ann Arbor, MI, USA) for 6 h at 37 °C before fluorescence was measured at 560/630 nm (both AB or NR) using the VictorX4.

### 3.5. Western Blot Analysis of MHV-68-Infected Cells

HFFs, Vero cells, COS-7, and MEFs were seeded in a 6-well plate. For each cell type, the cells were either left mock-infected or infected with MHV-68-Luc. Four d p.i., the cells were lysed in 100 µL, and two times 10 µL of each lysate was used to analyze the luciferase expression. The luciferase assay was performed as described for the MHV-68-Luc antiviral assay. To estimate the total protein amount per sample, a bicinchoninic acid assay (BCA) was performed by using a Pierce™ BCA Protein Assay Kit (Thermofisher, Rockford, IL, USA). For each sample, two times 10 µL of total protein lysate or a 1:10 dilution was used to determine the total protein amount. All steps were performed following the manufacturer’s protocol, and the colorimetric change was measured at 562 nm using the VictorX4. From the remaining lysate, a protein amount of 30 µg/mL was used for Western blot analysis of CDK8 expression, as previously described [35]. Western blot analysis was performed using a CDK8-specific antibody (A302-501A, Bethyl Laboratories, Montgomery, TX, USA) and a monoclonal β-actin antibody (A5441, Sigma-Aldrich, St. Louis, MO, USA) as a loading control. Detection was carried out using the appropriate HRP-conjugated secondary antibodies.

## 4. Conclusions

In the present study, we focused our analysis on specific assay systems to apply innovative readout measurements. This approach was intended to provide insight into the broad-spectrum antiherpesviral and non-herpesviral activity of recently identified CDK8 inhibitors. The assays comprised a variety of reporter-driven quantitative assessments, in particular, for four herpesviruses, i.e., EHV-1, HHV-6A, EBV, and MHV-68, and two non-herpesviruses, i.e., VV, and SARS-CoV-2. Interestingly, the results of the present study, together with the data published by our group before [35,38], provide a relatively wide spectrum of viruses analyzed for CDK8 inhibitor sensitivity so far (Table S1). The viruses showing antiviral effects against CDK8 inhibitors, as expressed by defined micromolar-to-nanomolar EC_50_ values, span members of the families *Herpesviridae* (species of α-, β-, and γ-herpesviruses), *Adenoviridae*, and *Polyomaviridae* and may include even more. On the other hand, two examples of *Coronaviridae* and *Poxviridae* did not show CDK8 inhibitor sensitivity. Concerning the possible role of CDK8 in viral replication, the current knowledge is rather limited, so that on the basis of our analyses, two branches of virus-supportive functions of CDK8 appear plausible. On the one hand, the fine modulation of transcription mediated by CDK activity in virus-infected cells is a very profound regulatory mechanism. In this regard, the replication efficiency of most human and animal viruses, if not all, behaves sensitive towards changes in the host transcription machinery, and obviously a number of viruses are dependent on the transcription factor CDK8. On the other hand, viral proteins may represent direct substrates of the CDK8 kinase and may thereby become regulated in a phosphorylation-mediated manner. This has been demonstrated for CDK7-specific phosphorylation of cytomegalovirus proteins before, and a similar situation may contribute to viral sensitivity towards CDK8 activity. Beyond these two regulatory mechanisms, even more CDK8-specific processes might have relevance for individual viruses, such as a virus-induced modulation of CDK8 activity. This may be conferred through the cyclin-binding property of viral proteins, as recently specified for HCMV [36,69,70,71], but this aspect still remains speculative. Thus, our experimental achievements were as follows: (i) CDK8 inhibitors proved to possess a very pronounced and relatively broad antiviral activity; (ii) the antiviral spectrum spanned in particular across certain subfamily members of α-, β-, and γ-herpesviruses; (iii) non-herpesviruses showed a relatively weak or no sensitivity; and thus (iv), CDK8 may represent an interesting target of host-directed drug candidates. Combined, the novel antiviral data substantiate the concept of a broad herpesvirus-supportive role of human CDK8 and its potential as a new target of refined pharmacological strategies.

## Figures and Tables

**Table 1:** Selected quantitative virus assay systems for comparative antiviral drug analysis.

Virus Classification	*Herpesviridae*				*Poxviridae*	*Coronaviridae*
	α (Animal)	β (Human)	γ (Human)	γ (Animal)	(DNA Genome, Human)	(RNA Genome, Human)
**Virus strain/reporter**	equine herpesvirus EHV-1-GFP	human herpesvirus 6 HHV-6A-GFP	Epstein–Barr virus P3HR-1	murine herpesvirus 68 MHV-68-Luc	vaccinia virus VV IHD-5	SARS-CoV-2 d6-YFP
**Host cells**	Vero, COS-7	HFF, J-Jhan	P3HR-1	Vero, COS-7, MEF, HFF	293T	Caco-2
**Incubation times**	3–5 d	10–14 d	10 d	4–7 d	2 d	30 h
**Readouts**	GFP fluorometry	plaque reduction assay, GFP fluorometry	qPCR	luciferase reporter assay	luciferase reporter assay	YFP fluorometry
**Reference drugs** *	GCV, CDV, BCV	BCV, CDV	TFA, TDP, AFV	BCV, GCV, FOS, ACV, LMV, MBV	BCV, CDV	MPV (EIDD-1931)
**CDK8 inhib.** **sensitivity** ^§^	strong	strong	intermediate	strong/variable	low	low

* Abbreviations: GCV, ganciclovir; CDV, cidofovir; BCV, brincidofovir; TFA, tenofovir alafenamide; TDP, tenofovir disoproxil; AFV, adefovir; FOS, foscarnet; ACV; aciclovir; LMV, letermovir; MBV, maribavir; MPV, molnupiravir (EIDD-1931, active metabolite of MPV). ^§^ The following categorization was based on the findings described by the Figures: strong, EC_50_ < 1 µM; intermediate, EC_50_ 1–10 µM; low EC_50_ > 10 µM; variable, EC_50_ dependent on cell type.

## Data Availability

The original contributions presented in this study are included in the article. Further inquiries can be directed to the corresponding author.

## Supplementary Materials

The following supporting information can be downloaded at: https://www.mdpi.com/article/10.3390/ph18101560/s1. Figure S1. Virus release of MHV-68-Luc-infected COS-7 cells. Table S1. Summarizing overview of all antiviral EC50 values of CDK8 inhibitors determined so far against human and animal pathogenic viruses [µM].
