# Supplementary material for: New Assay Systems to Characterize the Broad-Spectrum Antiherpesviral and Non-Herpesviral Activity of Cyclin-Dependent Kinase (CDK) 8 Inhibitors
Source: Pharmaceuticals (Basel). 2025 Oct 16;18(10):1560. doi: 10.3390/ph18101560 (PMC12567398; doi:10.3390/ph18101560)
Supplement: Supplementary file 1 [file pharmaceuticals-18-01560-s001.zip › pharmaceuticals-3910668-supplementary.pdf]

pharmaceuticals

*Supplementary Materials*

# New assay systems to characterize the broad-spectrum antiherpesviral and non-herpesviral activity of cyclin-dependent kinase (CDK) 8 inhibitors

**Debora Obergfäll [1], Friedrich Hahn [1,§], Jintawee Kicuntod [1], Christina Wangen [1], Melanie Kögler [1], Sabrina Wagner [1], Benedikt Kaufer [2], Manfred Marschall [1,*]**

[1] Harald zur Hausen Institute of Virology, Friedrich-Alexander-Universität Erlangen-Nürnberg (FAU), Erlangen, Germany
[2] Institute of Virology, Freie Universität Berlin, Berlin, Germany
§ Present address: Institute of Virology, Ulm University Medical Center, Ulm, Germany
* Correspondence: manfred.marschall@fau.de; Phone +49-9131-85-36096

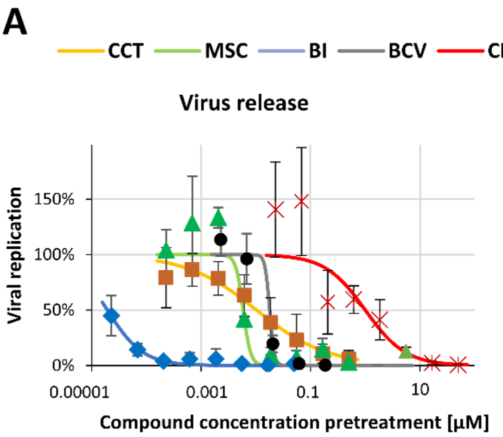


**B**

Antiviral mean $EC_{50}$ values based on luciferase expression [µM]

| Drug | $EC_{50}$ |
|---|---|
| CCT-251921 | 0.01 ± 0.02 |
| MSC-2530818 | 0.006 ± 0.0001 |
| BI-1347 | <0.00002 |
| BCV | 0.02 ± 0.004 |
| CDV | 1.0 ± 0.9 |

**Figure S1.** Virus release of MHV-68-Luc-infected COS-7 cells. **(A)** COS-7 cells were used for infection with MHV-68-Luc as described for experiments in Fig. 4. At the time point of 4 d p.i., under treatment with either CCT-151921, MSC-2530818, BI-1347, BCV or CDV, a volume of 5 µl of the culture supernatant of each well was transferred to newly seeded COS-7 cells. From these, luciferase assay was performed 3 d post-transfer to estimate the virus release from the drug-treated cells compared to DMSO, as performed in quadruplicates. **(B)** The calculation of antiviral effects ($EC_{50}$ values) was based on the Luc signal obtained from the release virus used for transfer infection

**Table S1.** Summarizing overview of all antiviral EC50 values [μM] of CDK8 inhibitors determined so far against human and animal viruses

| Compounds | β-herpesviruses | | | | | | | |
|---|---|---|---|---|---|---|---|---|
| | CMVs | | | | | | HHV-6A | |
| | HCMV | RhCMV | ChCMV | MCMV | RCMV | GPCMV | HHV-6A (J-Jhan) | HHV-6A (HFF) |
| CCT-151921 | 0.004 ± 0.002 | 0.003 ± 0.002 | 0.03 ± 0.02 | >0.04 | 0.02 ± 0.05 | 0.05 ± 0.08 | 39.0 ± 21.0 | 0.04 ± 0.02 |
| MSC-2530818 | 0.001 ± 0.0007 | 0.002 ± 0.001 | 0.02 ± 0.03 | 0.02 ± 0.02 | 0.003 ± 0.008 | 0.03 ± 0.05 | >50.0 | 0.3 ± 0.3 |
| BI-1347 | 0.0009 ± 0.0007 | 0.0004 ± 0.0005 | 0.01 ± 0.02 | 0.02 ± 0.01 | 0.002 ± 0.004 | n.d. | >50.0 | 0.3 ± 0.2 |

| Compounds | α-herpesviruses | | | | γ-herpesviruses | | | | | |
|---|---|---|---|---|---|---|---|---|---|---|
| | HSV-1 | VZV | EHV-1 (COS-7) | EHV-1 (HFF) | KSHV | EBV | MHV-68 (Vero) | MHV-68 (COS-7) | MHV-68 (HFF) | MHV-68 (MEF) |
| CCT-151921 | >50.0 | 28.0 ± 9.0 | 0.03 ± 0.03 | 0.02 ± 0.005 | 0.3 ± 1.0 | 2.5 ± 1.0 | 0.002 ± 0.001 | 0.0005 ± 0.0009 | 0.5 ± 0.3 | 0.01 ± 0.009 |
| MSC-2530818 | 42.3 ± 4.9 | >50.0 | 0.10 ± 0.6 | 0.8 ± 4.2 | >40.0 | 1.4 ± 0.6 | 0.002 ± 0.07 | 0.003 ± 0.004 | 1.8 ± 1.8 | 0.02 ± 0.02 |
| BI-1347 | >50.0 | 26.0 ± 10.0 | 0.005 ± 0.3 | 0.03 ± 0.03 | 0.3 ± 0.6 | 5.5 ± 4.2 | 0.0005 ± 0.00003 | 0.0001 ± 0.0001 | 0.04 ± 0.02 | 0.006 ± 0.002 |

| Compounds | non-herpesviruses | | | |
|---|---|---|---|---|
| | HAdV | JCPyV | SARS-CoV-2 | VV |
| CCT-151921 | 0.03 ± 0.03 | 3.7 ± 3.0 | 62.4 ± 18.5 | >50.0 |
| MSC-2530818 | 0.3 ± 0.3 | 0.1 ± 3.5 | 15.4 ± 6.4 | >50.0 |
| BI-1347 | 0.05 ± 0.1 | 0.7 ± 1.6 | 22.8 ± 3.2 | >50.0 |
